# Bupropion Maintenance Treatment in Refractory Bipolar Depression: A Case Report

**DOI:** 10.2174/1745017901713010043

**Published:** 2017-05-31

**Authors:** Julia Dehning, Heinz Grunze, Armand Hausmann

**Affiliations:** 1 Department of Medicine, Paracelsus Medical University, Salzburg, Austria; 2 Department of Medicine, Medical University - Psychiatry, Innsbruck, Austria

**Keywords:** Bipolar disorder, Bupropion, Depression, Guidelines, Maintenance treatment, TEAS

## Abstract

**Background:**

The optimal duration of antidepressant treatment in bipolar depression appears to be controversial due to a lack of quality evidence, and guideline recommendations are either vague or contradictive. This is especially true for second line treatments such as bupropion that had not been subject to rigourous long term studies in Bipolar Disorder.

**Case presentation:**

We report the case of a 75 year old woman who presented with treatment refractory bipolar depression. Because of insufficient response to previous mood stabilizer treatment and refractory depressive symptoms, bupropion was added to venlafaxine and lamotrigine. From there onwards, the patient improved continuously without experiencing deterioration of depression or a switch into hypomania. Our patient being on antidepressants for allmost four years experienced an obvious benefit from longterm antidepressant administration.

**Conclusion:**

Noradrenergic/dopaminergic mechanisms of action may play a more prominent role in bipolar depression, and may still be underused as a therapeutic strategy in the acute phase as well as in long-term maintenance in at least a subgroup of bipolar patients. There is still a lack of evidence from RCTs, but this case report further supports antidepressant long-term continuation and the usefulness of a noradrenergic/dopaminergic antidepressant in the acute and maintenance treatment of bipolar disorder.

## 1 INTRODUCTION

The appropiate duration of antidepressant treatment in bipolar depression remains under dispute. Older Bipolar Guidelines suggest an early tapering of the antidepressant, in a time range from 2 to 6 months [1-3]. However, these recommendations are mainly opinion- driven and little evidence-based, and do not take into consideration that a fair subgroup of treatment-refractory patients may need more time to recover. With this short time frame, residual symptoms are likely to persist and might give rise to an early relapse [4, 5]. In addition, the risk of developing a new depressive episode is greater with a depressive than a manic index episode [6]. Uncontrolled evidence suggests that early tapering of antidepressants within 6 months significantly increases the risk of relapse within one year [7]. In the absence of sound studies, there is no scientific evidence for tapering either at 2 or 6 months, but some opinion papers [8, 9] and guidelines [10, 11] suggest that antidepressants should be continued at least until full remission is achieved.

Whereas some placebo-controlled long term data are available for commonly used antidepressants, such as imipramine [12] or fluoxetine [13] in bipolar II depression, little is known about long term benefits and harm of other antidepressants in Bipolar disorder. Data from a one-year, double blind follow up of acute responders to add on venlafaxine, sertraline or bupropion (but with no placebo control) suggest maintenance of antidepressant effects for all three antidepressants without an increased risk of manic switches [14]. However, virtually nothing has been published about antidepressant effectiveness in Bipolar disorder for durations of treatment greater than one year, especially in treatment of refractory patients.

## 2 CASE PRESENTATION

We report the case of a 75 year old woman who presented in 2004 with treatment refractory bipolar depression. She was admitted to hospital for the first time after a suicide attempt in 1996. After being insufficiently responsive to multiple psychopharmacological strategies, including add-on and augmentation therapies, she underwent a first course of ECT in 1997. For recurrent episodes, she also received ECT in 2001, 2003 and 2004. Ten sessions of monthly maintenance ECT (right unilateral) were administered in 2003 and 2004, only leading to subsequent continuous deterioration of cognitive function without having a pronounced antidepressant effect. In 2001, she experienced her only hypomanic episode after administration of the tricyclic nortriptyline (100 mg/d). At this time, she was diagnosed as having bipolar III disorder according to a broader bipolar spectrum concept [15], but according to DSMV [16], her illness would now be classified as Bipolar II disorder. The patient had a family history for depression and bipolar disorder.

In 2004 she was prescribed tianeptine and pregabalin as a presumed mood stabilizer [17]. Shortly afterwards, she made a second suicide attempt. She subsequently had venlafaxine (150 mg/d) and lamotrigine (200 mg/d) implemented. After more than three months on this medication without noticeable change, she was then admitted to our specialized outpatient clinic for mood disorders in Innsbruck, Austria. She presented with residual depressive symptoms such as residual low mood and anxiety, fatigue, lack of energy, motor retardation and cognitive impairment. She was disabled to such a degree that she was not even able to dress appropriately and fulfilled stringent criteria for treatment of refractory bipolar depression [18, 19]. Because of insufficient response to previous mood stabilizer treatment, bupropion (300 mg/d) was added to the ongoing medication. From there onwards, the patient improved continuously. After 6 weeks, she fulfilled criteria for symptomatic remission [18], minor subsyndromal symptoms vanished over the next year and she remained well for four years (end of follow up and referral back to community treatment), without experiencing a depressive relapse or a switch into hypomania.

## 3 DISCUSSION AND CONCLUSION

Different from North America, bupropion still appears underused in other countries as a treatment of choice in bipolar depression, and with the only large randomized, placebo-controlled acute study as add-on to mood stabilizer having failed [20], it often receives a low grade of recommendation in some guidelines [21, 22], but not all [10, 23]. Previous open studies with bupropion, however, were suggestive of good antidepressant efficacy in bipolar depression [24-26], though in one study at the expense of treatment emergent affective switches [27]. Although becoming unresponsive to ECT as an indicator of potential treatment refractoriness, our patient did respond well to the addition of bupropion to the ongoing regimen with venlafaxine and lamotrigine. Bupropion as a norepinephrine and dopamine re-uptake inhibitor constitutes a unique antidepressant class of its own [28]. Dopamine is assumed to play a critical role in depressive symptoms like motor retardation, fatigue and leaden paralysis. Many treatment refractory depressive patients are thought to belong to a broader bipolar spectrum [29], and symptoms of fatigue and motor retardation appear more common in bipolar than unipolar depressives [30]. Enhancing dopaminergic transmission may be effective in a subgroup difficult to treat bipolar depressed patients [31, 32], and may be additionally beneficial in those with cognitive impairment [33]. In line with this assumption, tranylcypromine, a non-reversible, non-selective monoamineoxydase inhibitor (enhancement of all three biogenic amines), was tested in an open, randomized, yet underpowered study *vs.* lamotrigine in the treatment of refractory bipolar depressed patients. 70% responded to tranylcypromine without switch compared to 30.8% on lamotrigine, a finding that almost reached significance (*p* = .08) [34]. In contrast to common expectations, there was also no difference in frequency and severity of side effects.

Depite their frequent use in clinical practice [35], there is also sparse published evidence on maintenance therapy with antidepressants as a group in bipolar disorder. An older review detected only 7 controlled studies [36], all comparing the tricyclic imipramine with placebo or lithium. The essence of these studies was that antidepressants do not protect against a relapse and that they put patients on risk of more switches and a higher episode frequency. The methodological limitations of these studies had been elaborated quite diligently [37], and the evidence-based Guideline of the British Association of Psychopharmacology (BAP) came in 2009 to the conclusion that the evidence is insufficient to recommend antidepressant discontinuation as a general principle [38]. More recent, however, a controlled maintenance study of Ghaemi *et al.* [39], was supportive of long term benefits of antidepressant treatment in addition to mood stabilizers in bipolar disorder, except of rapid cycling patients. Despite the conflicting evidence, a recent expert panel agreed that antidepressant continuation might be helpful in selected patients [40]. Our patient had an obvious benefit from long term antidepressant administration, she expressed herself as feeling for the first time “as a human being” after a period of almost 4 years of chronic depression necessitating several courses of ECT without persisting benefits.

Furthermore, she did not only recover from depression, but also remained free from emerging manic symptoms. Due to the assumed risk of inducing a hypomanic switch, some older guidelines had discouraged the use of antidepressants in general (*e.g.*, [1, 3]). However, none of the more recent meta analyses of RCTs of antidepressants in bipolar depression have found a signal for an increased risk of treatment-emergent affective switches (TEAS) [41-43], with the reservation that in the vast majority of studies, antidepressants had been administered together with an antimanic agent. This has now been reflected both in more recent consensus statements [40] and guidelines (*e.g.*, [10, 11, 23, 44, 45]) which are clearly less restrictive concerning antidepressant, at least for the short and intermediate term use.

## LIST OF ABBREVIATIONS

BAP: = British Association of Psychopharmacology
ECT: = Electroconvulsive Therapy
DSM: = Diagnostic and Statistical Manual
TEAS: = Treatment-Emergent Affective Switch
